# Diagnostic value of procalcitonin for identifying the etiology of lower respiratory tract infections: a systematic review and meta-analysis

**DOI:** 10.1186/s12879-025-12426-9

**Published:** 2025-12-25

**Authors:** Chang-Yang Lin, Xun Zhou, Gai-Gai Li, Ju Qiu, Dan Li, Qi-Yuan Pang

**Affiliations:** 1 Department of Pulmonary and Critical Care Medicine, The Second Affiliated Hospital of Guizhou University of Traditional Chinese Medicine, Guiyang, China; 2 Department of Nursing, Guiyang Hospital of Stomatology, Guiyang, China

**Keywords:** Procalcitonin, Microbiology, Meta-analysis, Diagnosis, Biomarker

## Abstract

**Background:**

Differentiating pathogens of lower respiratory tract infections(LRTIs) is challenging. This study aims to determine whether procalcitonin(PCT) level could distinguish different pathogens in adults with LRTIs.

**Methods:**

We searched the PubMed, EMBASE, and the Cochrane Central Register of Controlled Trails that identified all relevant diagnostic accuracy studies. Studies were assessed for reporting of diagnostic accuracy, relevance and quality. Data were extracted for meta-analysis.

**Results:**

18 trials involving 3174 patients were included. For differentiating bacteria from viruses, pooled sensitivity, specificity, positive likelihood ratio(+LR), negative likelihood ratio(−LR) and diagnostic odds ratio(DOR) for PCT(0·5ug/L) were 0.49(95%CI, 0.33–0.66), 0.80(95%CI,0.65–0.90), 2.48(95%CI,1.75–3.50), 0.63 (95%CI, 0.52–0.77) and 3.93(95%CI,2.87–5.37), respectively. For differentiating typical pathogens from atypical pathogens, pooled sensitivity, specificity, +LR, -LR and DOR for PCT(0·5ug/L) were 0.60(95%CI,0.41–0.76), 0.81(95%CI,0.61–0.92), 3.09(95%CI,1.80–5.32), 0.50(95%CI,0.37–0.68) and 6.20(95%CI,3.76–10.21), respectively.

**Conclusions:**

The results of this meta-analysis showed that PCT has moderate diagnostic accuracy for lower respiratory tract pathogens such as bacteria, viruses, and atypical pathogens.

## Introduction

Lower respiratory tract infections(LRTIs), including community acquired pneumonia(CAP), hospital acquired pneumonia(HAP), bronchitis, bronchiolitis and acute exacerbation of chronic obstructive pulmonary disease, are the fourth-leading causes of mortality worldwide. LRTIs are caused by a variety of pathogens, such as bacteria, viruses, mycoplasma, and fungi. LRTIs are prevalent and potentially lethal, which has been reported to cause 2.60 million deaths in 2019 [1, 2]. For infectious diseases, it is important to identify pathogenic microorganisms to choose the appropriate therapeutic regimen. However, pathogenic determination of LRTIs is typically challenging based on clinical assessment alone. Currently, the gold standard for identifying pathogens is culture, which is time-consuming and has low sensitivity. In addition, immunological assays and molecular biology technologies have also been used to detect specific pathogens [3, 4]. Although various assays exist, rapid and accurate identification of pathogens is still difficult [5].

Procalcitonin(PCT) is a prohormone produced by parafollicular cells of the thyroid, which is significantly increased in bacterial infections [6]. In most viral infections, the synthesis of PCT is inhibited, leading to bacterial specificity of PCT. [7] PCT has been widely used in clinical practice [8]. PCT is a useful method for guiding the initiation and duration of antibiotic treatment for LRTIs [9, 10]. However, whether PCT can distinguish pathogenic microorganism in LRTIs is controversial.

Therefore, the aim of the present systematic review and meta-analysis was to determine whether PCT levels could distinguish different pathogens in adults with LRTIs.

## Results

### Search results

**Fig. 1:**
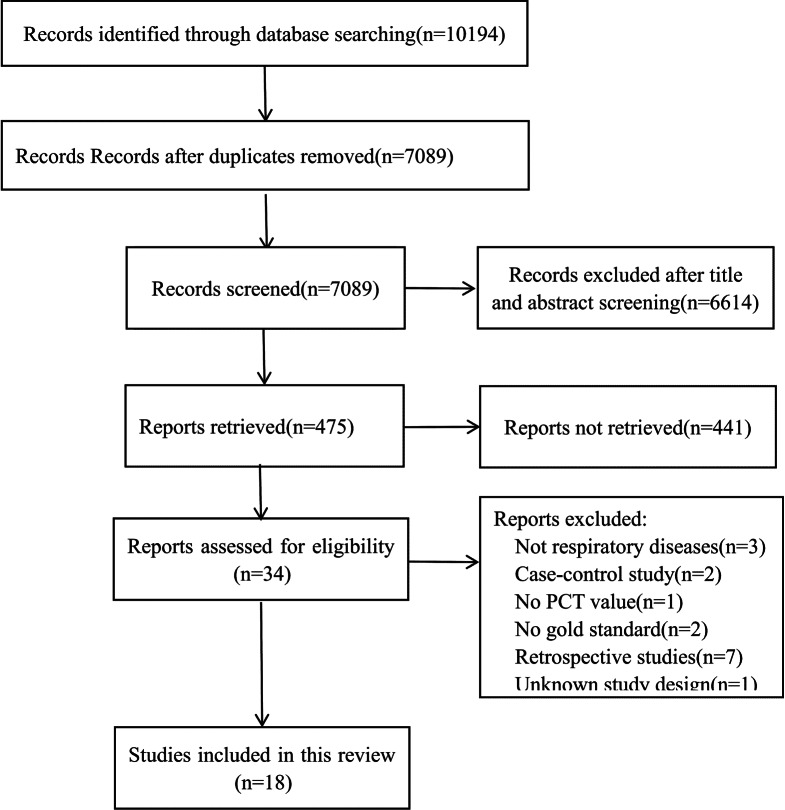
PRISMA flow diagram

The literature screening flow diagram is detailed in Fig. 1. A total of 10194 studies were retrieved. Subsequently, 18 studies were included in our analysis, including a total of 3174 patients [11–28].

### Trial characteristics

Table 1 shows the characteristics of the included trials. For the geographic regions, 9 trials were conducted in Europe, 5 in Asia，and 4 in the United States. For the disease types, 13 trials involved with CAP, 4 trials involved patients with LRTI, and 1 trial involved patients with AECOPD. Three trials were multi-center studies, and the remaining 15 trials were single-center studies. Among all 3174 patients, 1263 patients were in the bacterial group, 1628 were in the viral group, and 283 were in the atypical group. These patients included 1082 ICU patients, 482 ED patients, and 1610 ward patients. All studies described diagnostic cutoff thresholds for PCT, ranging from 0.1 µg/L to 2 µg/L.

**Table 1:** Characteristic of included studies in this meta-analysis

Author/Year	Location	Study design	Diseases	Gold standard	Detection rate	Size	PCT cutoff(ug/L)
Hedlund/2000	Sweden	Prospective	CAP	blood and sputum culture, enzyme immunoassay	37.5%	36	0.50
Masia/2005	Spain	Prospective	CAP	blood and sputum culture, urinary antigens, enzyme immunoassay	48.6%	90	0.50
Boussekey/2005	France	Prospective	CAP	lower respiratory tract secretions and blood culture	43.6%	48	2.00
Jereb/2006	Slovenia	Prospective	CAP	sputum culture, urinary antigens, enzyme immunoassay	Not mentioned	30	0.50
Ip/2007	China	Prospective	LRTI	blood and sputum culture, indirect immunofluorescence assays	Not mentioned	267	0.10 0.50
Hirakata/2008	Japan	Prospective	CAP	blood and sputum culture, urinary antigens	45.5%	40	0.50
Daubin/2009	France	Prospective	COPD+CAP	Sputum, tracheal aspirates and blood culture, indirect immunofluorescence assays, urinary antigens, RT-PCR and PCR	44.1%	15	0.50
Falsey/2012	America	Prospective	AECOPD	blood and sputum culture, influenza antigen testing	23.8%	57	0.25
Kasamatsu/2012	Japan	Prospective	CAP	sputum and pharyngeal aspirates culture, urinary antigens, enzyme immunoassay	51.3%	116	0.50
Menendez/2012	Spain	Prospective	CAP	sputum, pharyngeal aspirates and blood culture, urinary antigens, enzyme immunoassay, detection of viral nucleic acids	40.7%	236	0.50
Espana/2012	Spain	Prospective	CAP	blood and sputum culture, urinary antigens, enzyme immunoassay	44.5%	153	0.15
ten Oever/2012	Netherlands	Prospective	LRTI	blood and sputum culture, urinary antigens, PCR	16.4%	56	0.50
Musher/2013	America	Prospective	CAP	blood and sputum culture, urinary antigens, PCR	39.4%	102	0.25
Choi/2014	Korea	Prospective	CAP	endotracheal aspirates culture, urinary antigens, PCR, RT-PCR	18.1%	47	0.70
Suarez/2015	America	Prospective	LRTI	blood and sputum culture, PCR	46.6%	55	0.25
Rodriguez/2016	Spain	Prospective	CAP	blood and sputum culture, urinary antigens, enzyme immunoassay, PCR	Not mentioned	972	0.50
Self/2017	America	Prospective	CAP	blood and sputum culture, urinary antigens, RT-PCR	37.2%	645	0.50
Duan/2021	China	Prospective	LRTI	sputum, blood and tracheal aspirates culture, urinary antigens, RT-PCR	26.1%	209	0.18

CAP: Community-acquired Pneumonia; AECOPD: Acute Exacerbation of Chronic Obstructive Pulmonary Disease; LRTI: Lower Respiratory Tract Infection; RT-PCR: Reverse transcriptase polymerase chain reaction; PCR: Polymerase chain reaction

### Quality assessment

**Fig. 2:**
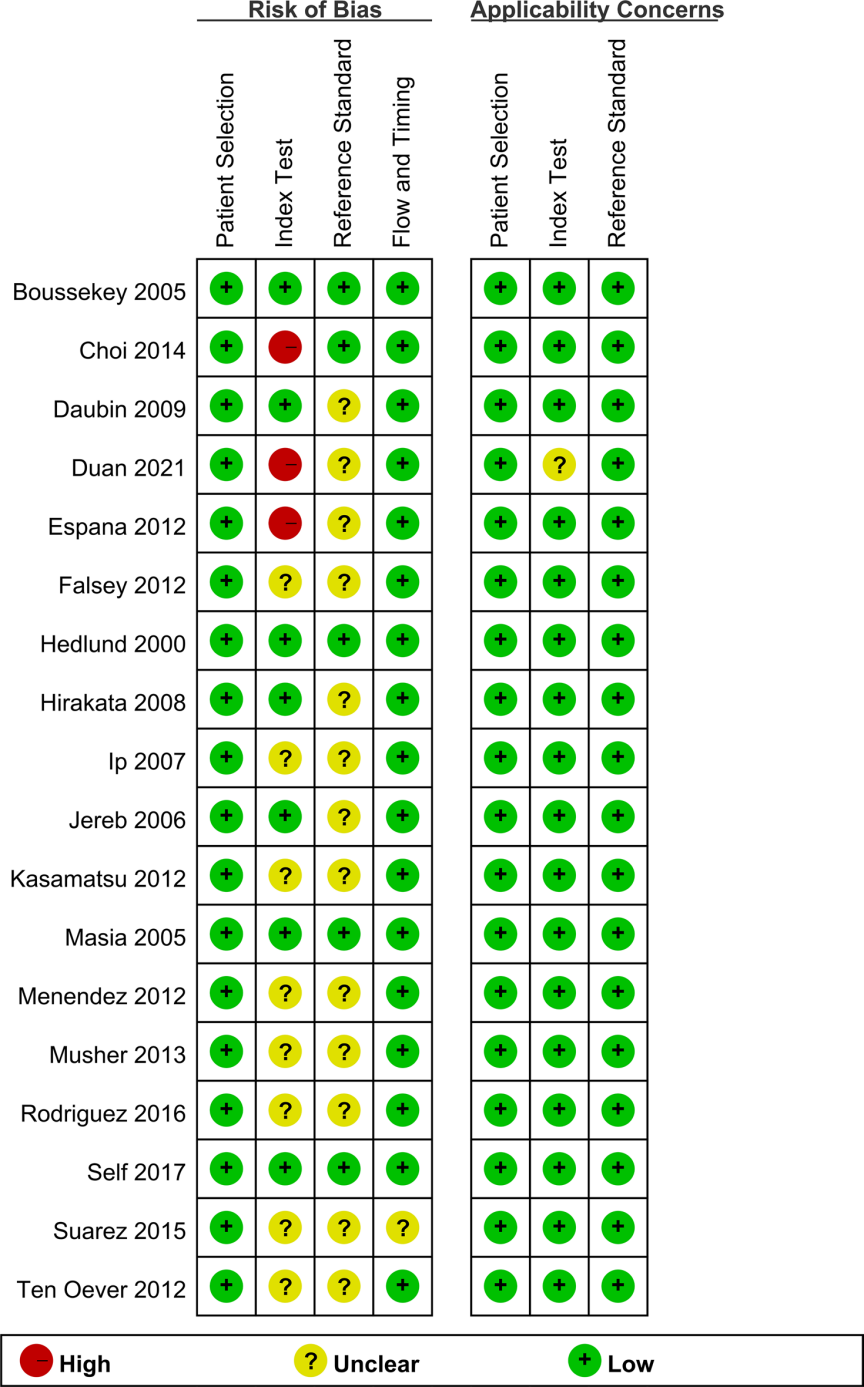
QUADAS-2 risk of bias and applicability concerns summary

The risk of bias assessment using the QUADAS-2 criteria is summarized in Fig. 2. Studies that illustrated unclear risk owing to the absence of blinding for reference standards and index tests, whereas all other studies were at low risk. High risk is represented by a red circle, low risk by a green circle, and unclear risk by a yellow circle.

### PCT distinguishes between bacteria and viruses

**Fig. 3:**
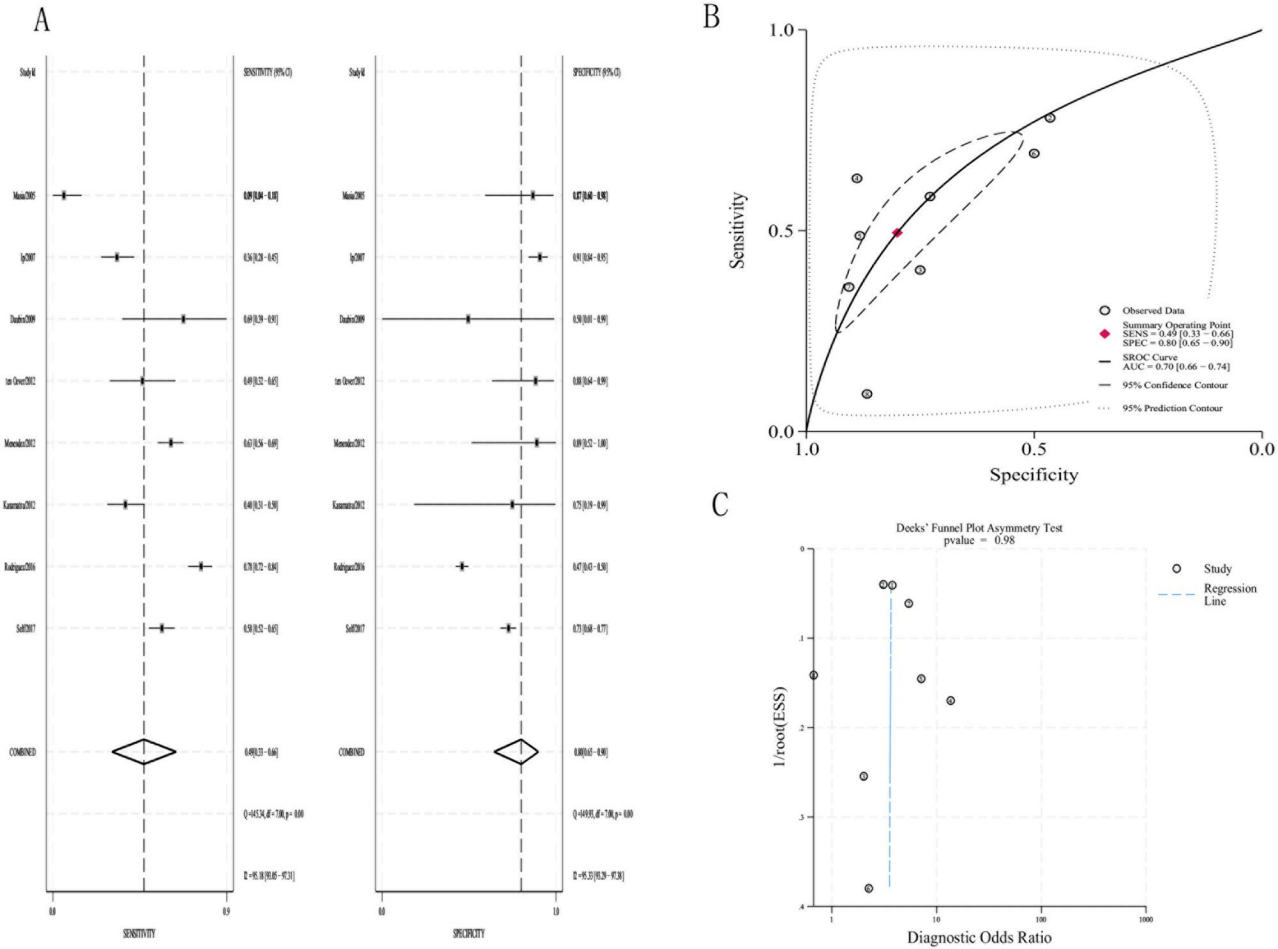
(**a**)The pooled sensitivity and specificity of PCT(0.5ug/L) to distinguish bacteria from virus; (**b**)summary receiver operator curve(SROC) for the diagnosis; (**c**)Deeks’ funnel plot asymmetry test

According to the 8 studies using a PCT cutoff of 0.5 µg/L, the pooled

sensitivity and specificity for LRTIs were 0.49 (95% CI, 0.33–0.66) and 0.80 (95% CI, 0.65–0.90), respectively(Fig. 3A). The pooled positive likelihood ratio(+LR), negative likelihood ratio(−LR) and DOR (diagnostic odds ratio) were 2.48(95%CI, 1.75–3.50), 0.63(95%CI, 0.52–0.77) and 3.93(95%CI, 2.87–5.37), respectively.(Table 2) The area under SROC curve was 0.70 (95% CI, 0.66–0.74)(Fig. 3B). The Deeks funnel plot was symmetric (*p* = 0.98), showing that bias and systematic heterogeneity were not significant (Fig. 3C).

### PCT distinguishes between typical and atypical pathogens

**Fig. 4:**
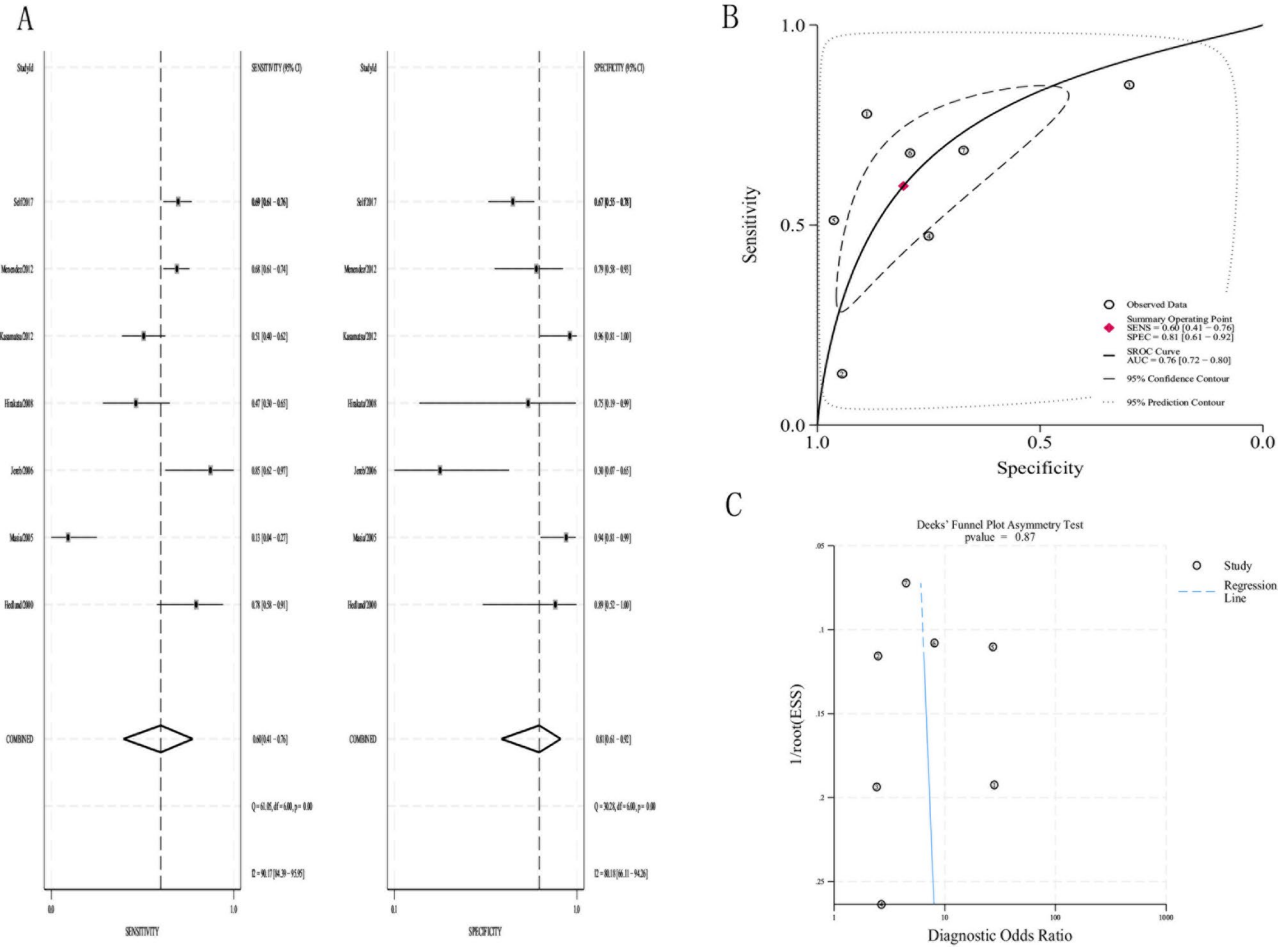
(**a**)The pooled sensitivity and specificity of PCT(0.5ug/L) to distinguish typical from atypical pathogens; (**b**)Summary receiver operator curve(SROC) for the diagnosis; (**c**)Deeks’ funnel plot asymmetry test

According to the 7 studies using a PCT cutoff of 0.5 µg/L, the pooled sensitivity and specificity were 0.60 (95% CI, 0.41–0.76) and 0.81(95% CI, 0.61–0.92), respectively(Fig. 4A). The pooled +LR, -LR and DOR were 3.1(95%CI, 1.8–5.3), 0.50(95%CI, 0.37–0.68) and 6.20(95%CI, 3.76–10.21), respectively.(Table 2) The area under SROC curve was 0.76 (95% CI, 0.72–0.80), which is a moderate result(Fig. 4B). The Deeks funnel plot was symmetric (*p* = 0.87) (Fig. 4C).

### Subgroup analysis

We performed the following subgroup analysis for the variables “disease, specific virus (influenza), disease severity” (Table 2).

**Table 2:** Subgroup analysis of PCT for LRTIs

Subgroup	Number of studies	PCT cutoff(ug/L)	Sensitivity (95% CI)	Specificity (95% CI)	+LR (95% CI)	-LR (95% CI)	DOR (95% CI)
**PCT for distinguishing bacteria and viruses**							
All	8	0.5	0.49(0.33-0.66)	0.80(0.65-0.90)	2.48(1.75-3.50)	0.63(0.52-0.77)	3.93(2.87-5.37)
CAP	6	0.5	0.52(0.31-0.73)	0.76(0.55-0.89)	2.15(1.53-3.04)	0.63(0.48-0.83)	3.42(2.41-4.83)
influenza	4	0.5-1.5	0.75(0.65-0.83)	0.65(0.46-0.79)	2.13(1.35-3.35)	0.38(0.26-0.56)	5.58(2.64-11.83)
non-ICU	5	0.5	0.42(0.23-0.61)	0.85(0.73-0.97)	1.90(1.23-2.94)	0.75(0.55-1.02)	2.54(1.25-5.16)
All	5	0.25	0.51(0.35-0.67)	0.82(0.65-0.92)	2.88(1.53-5.44)	0.60(0.45-0.80)	4.84(2.20-10.66)
**PCT for distinguishing typical and atypical pathogens**							
All	7	0.5	0.60(0.41-0.76)	0.81(0.61-0.92)	3.09(1.80-5.32)	0.50(0.37-0.68)	6.20(3.76-10.21)

+LR: positive likelihood ratio; -LR: negative likelihood ratio; DOR: diagnostic odds ratio

## Discussion

This systematic review and meta-analysis revealed that PCT has only a moderate diagnostic accuracy on determining which pathogen is responsible for LRTIs, demonstrating limited sensitivity, specificity, +LR and -LR. Subgroup analysis suggested equally low sensitivity and high specificity, regardless of disease classification or severity. Therefore, it could not serve as an indicator in the initial therapeutic decision for patients with LRTIs.

PCT has been widely used as a serum biomarker in the field of infection diagnosis in recent years. Research has revealed that PCT is a useful diagnostic indicator for sepsis and postoperative infection [29–31]. However, the diagnosis in the above studies relies on clinical judgment rather than microbiological diagnosis. To the best of our knowledge, this systematic review and meta-analysis is the first to investigate the diagnostic accuracy of PCT for lower respiratory pathogens.

For LRTIs, PCT-guided antibiotic therapy based on serial PCT measurements could significantly reduce antibiotic exposure [32, 33]. It seems that PCT can effectively distinguish lower respiratory tract pathogens. Based on previous research, we selected PCT levels of 0.25 µg/L and 0.5 µg/L as thresholds for presenting our results. However, some published studies explored that optimal threshold have greater diagnostic value. For example, the studies by Ingram et al. [34] and Cuquemelle et al. [35] used a cutoff of 0.8 µg/L PCT to differentiate mixed bacterial infections from influenza viral infections, with diagnostic accuracies of 0.88 and 0.90, respectively.

To our knowledge, little research has focused on distinguishing atypical bacteria from typical bacteria. Most of these studies revealed that PCT values for patients with atypical bacteria were more similar to those with viruses than typical bacteria, particularly for Mycoplasma and Chlamydophila [13, 20, 21, 27]. Usually, most studies merged typical bacteria and atypical bacteria into one group. Therefore, it may have a negative impact on the value of PCT in identifying bacteria and viruses.

PCT has improved other traditional biomarkers, including count of white blood cells(WBC) and C-reactive protein(CRP). In a 2021 systematic review, Vasavada et al. [36] has found PCT is a better marker to predict postoperative infectious, compared to CRP(0.85 vs 0.77). The systematic review by Tan et al. [37] showed the diagnostic accuracy of PCT is higher than CRP for sepsis(0.85 vs 0.73). Therefore, although our findings demonstrated PCT is not recommended for use as a single diagnostic tool, it remains a better biomarker. Moreover, there are some studies did not use PCT as a single indicator. Ahn et al. [38] and ten Oever et al. [22] reported that the combination of PCT and CRP can improve diagnostic accuracy. Espana et al. [18] reported that PCT levels can increase the diagnostic efficacy of CURB-65.

This study was subject to a number of limitations. First, we detected substantial heterogeneity between studies which could not be exclude [39]. Second, the studies differed in several ways(e.g. study design, disease spectrum and methodological quality). Finally, all included studies utilized traditional microbiological testing methods as the gold standard with low detection rates, especially for rare and atypical pathogens. This will result in the pooled sensitivity and specificity not reflecting the true performance of the test. Metagenomic next-generation sequencing (mNGS), an independent, unbiased, and hypothesis-free approach, has served as a new diagnostic tool for respiratory infections in recent years [40]. Compared to traditional methods, mNGS can provide a comprehensive view of pathogens and improve detection rates. Thus, future research on this topic should be directed at prospective trials on the diagnostic ability of procalcitonin in patients determined by mNGS.

## Conclusion

The results of this meta-analysis showed that PCT detection of LRTIs has moderate diagnostic accuracy for lower respiratory tract pathogens such as bacteria, viruses, and atypical pathogens. It seemed that PCT might not be sufficient to distinguish lower respiratory pathogens alone and could not be routinely used in the initial therapeutic decision for patients with LRTIs. Therefore, further randomized clinical trials are needed to evaluate the impact of the use of PCT in the decision about antibiotic therapy on the prognosis of patients.

## Method

This systematic review and meta-analysis was performed strictly on the basis of the Preferred Reporting Items for Systematic Reviews and Meta-analyses(PRISMA) statement [41]. A protocol for this study was published in PROSPERO (registration number CRD42024543764).

## Data sources and searches

We searched the PubMed, EMBASE and Cochrane Central Register of Controlled Trials databases for articles published through April 2024 with no language restrictions. The search strategies applied the following key words: “LRTI” or “low respiratory tract infection” or “low respiratory tract infections” or “pneumonia” or “bronchitis” or “COPD” or “chronic obstructive pulmonary diseases” stands for disease, “procalcitonin” or “calcitonin precursor polyprotein” or “calcitonin-1” or “calcitonin 1” or “calcitonin related polypeptide alpha” or “PCT” represents target index. Additional data sources were examined, including conference proceedings and the reference lists of relevant studies. All databases were checked daily for newly updated studies.

## Study selection

Studies fulfilling the following selection criteria were included in this meta-analysis: (1) the type of study design were randomized controlled trials, cohort studies, observational, and prospective studies; (2) studies in which the serum PCT level was used to diagnose LRTIs; (3) studies in which we could indirectly or directly obtain a 2 × 2 table of PCT diagnoses for LRTIs; (4) the study population was adult patients; and (5) the reference standard for the diagnosis of LRTIs in this study were as follows:①new or progressive lung infiltration. ②temperature > 38^°^C or < 36.5^°^C,WBC count > 12 × 10^9^/L or < 4 × 10^9^/L,purulent endotracheal aspiration or sputum.

Trials were excluded if they met the following criteria: (1) the age of the subjects was < 19 years, (2) the type of study design was a case-control studies or experimental animal study, or(3) the trial was published as an abstract only.

## Data collection process

Two authors independently screened the literature according to the inclusion and exclusion criteria, and the relevant data were extracted into predesigned data collection forms. Any discrepancies between two reviewers were identified in a reconciliation process by a third reviewer and were subsequently reconciled among the extractors. The following data were collected from each study: author, year, location, study design, sample size, detection method, gold standard, PCT cutoff value, sensitivity (SN), specificity (SP), true positive (TP), false positive (FP), false negative (FN), and true negative (TN).

## Qualitative assessment

We used the Quality Assessment of Diagnostic Accuracy Studies tool (QUADAS-2) to evaluate the quality of each selected study provided by Review Manager software(RevMan 5.3), and any disagreements were resolved by consensus [42].

## Statistical analysis

This systematic review undertook an initial descriptive analysis of the included studies. A bivariate random-effects model was used to calculate pooled sensitivity, specificity, +LR, -LR and DOR. Summary statistics with 95% confidence intervals(CIs) were used to calculate pooled sensitivity and specificity. A symmetric summary receiver operating characteristic (SROC) curve was plotted with a 95% confidence contour and area under the curve (AUC). The heterogeneity was calculated with the I^2^ test. Heterogeneity among the studies was considered to be present if I^2^ was greater than 50%. The Deeks funnel plot was used to assess for bias. All data analyses were performed using the MIDAS module for STATA(v0.14) software.

## Data Availability

All data generated or analyzed during this study are included in this published article.
